# A Test and Extension of Lane and Terry’s (2000) Conceptual Model of Mood-Performance Relationships Using a Large Internet Sample

**DOI:** 10.3389/fpsyg.2017.00470

**Published:** 2017-04-18

**Authors:** Andrew M. Lane, Peter C. Terry, Tracey J. Devonport, Andrew P. Friesen, Peter A. Totterdell

**Affiliations:** ^1^Research Centre for Sport Exercise Performance, University of WolverhamptonWalsall, UK; ^2^University of Southern Queensland, Toowoomba, QLDAustralia; ^3^Department of Psychology, University of Sheffield, SheffieldSheffield, UK

**Keywords:** emotion, affect, concentration, mood, depression, dejection

## Abstract

The present study tested and extended Lane and Terry (2000) conceptual model of mood-performance relationships using a large dataset from an online experiment. Methodological and theoretical advances included testing a more balanced model of pleasant and unpleasant emotions, and evaluating relationships among emotion regulation traits, states and beliefs, psychological skills use, perceptions of performance, mental preparation, and effort exerted during competition. Participants (*N* = 73,588) completed measures of trait emotion regulation, emotion regulation beliefs, regulation efficacy, use of psychological skills, and rated their anger, anxiety, dejection, excitement, energy, and happiness before completing a competitive concentration task. Post-competition, participants completed measures of effort exerted, beliefs about the quality of mental preparation, and subjective performance. Results showed that dejection associated with worse performance with the no-dejection group performing 3.2% better. Dejection associated with higher anxiety and anger scores and lower energy, excitement, and happiness scores. The proposed moderating effect of dejection was supported for the anxiety-performance relationship but not the anger-performance relationship. In the no-dejection group, participants who reported moderate or high anxiety outperformed those reporting low anxiety by about 1.6%. Overall, results showed partial support for Lane and Terry’s model. In terms of extending the model, results showed dejection associated with greater use of suppression, less frequent use of re-appraisal and psychological skills, lower emotion regulation beliefs, and lower emotion regulation efficacy. Further, dejection associated with greater effort during performance, beliefs that pre-competition emotions did not assist goal achievement, and low subjective performance. Future research is required to investigate the role of intense emotions in emotion regulation and performance.

## Introduction

A wealth of empirical and anecdotal evidence indicates that emotions influence thoughts and actions, and that preparation for any type of performance typically involves attempts to regulate emotions (Lazarus, 2000; Baumeister et al., 2007; Hanin, 2010; Gross, 2015). Although people seem to intuitively understand the emotion construct, it remains difficult to distinguish from related constructs such as mood and affect from both theoretical and measurement perspectives (Baumeister et al., 2007; Lane and Terry, 2016). Emotions are generally seen as short in duration, influencing behavior, and related to specific antecedents (Lazarus, 2000) whereas moods are more enduring, diffuse, and lack a specific antecedent (Lane and Terry, 2000). In the present study, we chose to use the term emotion because we examined feelings in a specific context in relation to achieving a particular goal.

As a theoretical basis, we used a circumplex model of emotion that distinguishes between pleasant and unpleasant feelings, and between high and low activation levels (Larsen and Diener, 1987). Both pleasant and unpleasant high-activation emotions, such as excitement and anxiety, are commonly experienced in competitive contexts when important goals are pursued, although the influence of such emotions on performance is neither linear nor consistent (Beedie et al., 2000; Lane and Terry, 2000; Tamir, 2009, 2015; Hanin, 2010; Lane et al., 2012). Emotions influence action but how that occurs is determined by individual and situational factors (e.g., personal goals, previous experience, or task demands). It is apparent that people prefer to feel emotions they believe will help them achieve their goals, and try to regulate their feelings accordingly (Baumeister et al., 2007; Tamir, 2009; Hanin, 2010).

In terms of explaining inconsistent emotion-performance relationships, evolutionary psychologists argue that emotions function to provide situational information and that all emotions can be helpful or harmful in achieving goals regardless of intensity (Baumeister et al., 2007; Nesse and Ellsworth, 2009). For example, anger (triggering an approach action) evolved to help the human species survive in the physically competitive environment encountered by our predecessors and thus where action is needed anger might be useful (Tamir, 2009, 2015). On the other hand, anger might inhibit goal achievement if it associates with self-blame and the belief that investing effort is futile. Similarly depression, which is characterized as an unpleasant low-activation emotion, may functionally signal that resources (e.g., effort, attention, or time) need to be preserved for new goals or may be dysfunctional for tasks that require high activation such as those involving movement or rapid thinking (Nesse and Ellsworth, 2009).

Gross (2015) argued that the context in which emotions are experienced and the goals to which they relate, influence the direction of emotion-performance relationships. Tamir (2009) demonstrated that anger was helpful in a confrontation task where increased arousal and a resultant narrow attentional style were beneficial. Conversely, low intensity pleasant emotions such as happiness and calmness associate positively with tasks requiring creative thinking (Fredrickson, 2013). Baumeister et al. (2007) argued that individual and contextual factors should be considered in the process by which people develop beliefs about the influence of emotions on thoughts and behavior. They argued that emotions provide “learning rules” wherein people use previous emotional experiences to guide future behavior. If an individual felt angry and those feelings coincided with success, then the individual might attempt to increase feelings of anger in a future similar goal endeavor. Considerable research in sport psychology has supported the notion that athletes can learn to interpret unpleasant emotions such as anxiety and anger as helpful for performance (e.g., Robazza and Bortoli, 2007; Jones et al., 2009; Hanin, 2010).

**FIGURE 1:**
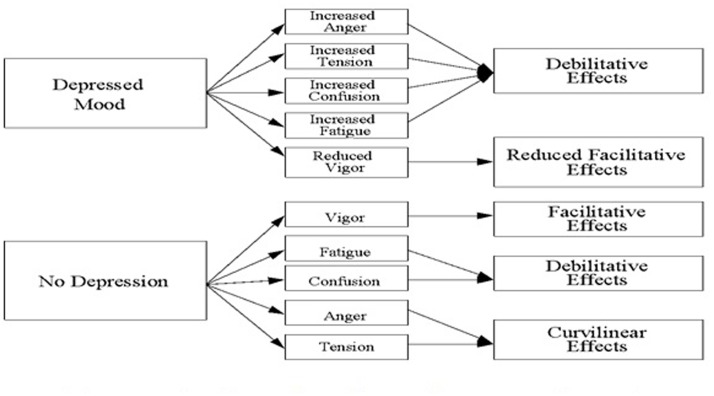
**A conceptual model to predict performance from pre-competition mood (Source: Lane and Terry, 2000, p. 24)**.

Despite the demonstrated functionality of some emotions for performance, identifying the exact circumstances under which an emotion will be helpful or harmful is challenging. Rather than investigating discrete emotions, such as examining anxiety in isolation from related emotions such as anger and dejection, Lane and Terry (2000) proposed that researchers and practitioners should consider combinations of emotions (**Figure 1**). Focusing on anger and tension (high-activation unpleasant emotions), Lane and Terry proposed that these two emotions are harmful to performance when experienced with other negative emotions, particularly depression. By contrast, the same two emotions can assist performance when experienced independently of depression. Their model draws on theory suggesting that emotions are informational in that they influence the interpretation of situational factors and personal resources to cope (Schwarz, 2011). Depression, in the context of Lane and Terry’s model, is characterized by feelings of unhappiness and dejection, and is typified by the recall of previous negative outcomes. As used in the model, the term depression describes a non-clinical emotional state, which could be synonymously labeled sadness (Lane et al., 2004) or dejection (Jones et al., 2005).

Lane and Terry (2000) proposed four hypotheses; first, that participants reporting symptoms of depression would tend to simultaneously report higher scores for anger, confusion, fatigue, and tension but lower scores for vigor. This hypothesis has been supported in all subsequent tests of the model (see Lane and Terry, 2016). The second hypothesis, that intercorrelations among discrete emotions would be stronger among those reporting symptoms of depression, has also been widely supported (Lane and Terry, 2016). The third hypothesis is that vigor facilitates performance whereas confusion and fatigue debilitate performance, regardless of the presence or absence of depression. When within-subject designs have been used to test this hypothesis and performance assessed using self-referenced measures sensitive to small performance variations, emotions have been shown to be predictive of performance (Lane and Terry, 2016). Research typically shows support for the positive effects of vigor on performance regardless of depression but less consistent performance effects for confusion and fatigue (Lane and Terry, 2016). The fourth hypothesis is that anger and tension tend to facilitate performance when experienced independently of depression, whereas they tend to debilitate performance when experienced in conjunction with depression. Lane and Terry (2000) proposed that when individuals feel depressed, angry, and tense they tend to perform poorly, whereas when they feel moderately angry and tense but not depressed they tend to perform well (Lane et al., 2001). Lane and Terry (2000) also proposed that anger and tension would show a curvilinear relationship with performance, arguing that over arousal would hinder performance. In asserting the pivotal position of depression, Lane and Terry (1999) tested whether anxiety exerted a similar influence. They found that anxiety did not associate with large differences in other emotions, supporting the notion that depression is a key variable when examining emotion-performance relationships.

The Lane and Terry (2000) model has previously been evaluated exclusively using the Profile of Mood States (POMS; McNair et al., 1971) or derivatives such as the Brunel Mood Scale (BRUMS; Terry et al., 1999, 2003), both of which assess five unpleasant states (anger, confusion, depression, fatigue, tension) and only one pleasant state (vigor). There has been much debate about the measurement of affective dimensions. Jones et al. (2005) argued that fatigue is a physiological state and confusion is a cognitive state, and excluded both from their emotion inventory. In the development of their model, Lane and Terry (2000) emphasized the conceptual distinction between happiness and vigor, noting that for tasks requiring high levels of arousal, happiness might hinder performance, whereas vigor would tend to generate greater effort to attain performance goals, particularly for tasks requiring physical exertion. Further, happiness is proposed to be associated with superficial processing of information which can have negative performance effects (Sinclair and Mark, 1992). To date, little research has compared the effects on performance of low-activation and high-activation pleasant emotions, such as happiness and vigor. Thus, it is not known whether the Lane and Terry model would be supported using valence-balanced measures such as the Sport Emotion Questionnaire (SEQ; Jones et al., 2005). The SEQ was selected for use in the present study as the underlying dimensions (anger, anxiety, dejection, excitement, and happiness) were identified from qualitative studies that sought to classify meaningful pre-competitive emotions. In the present study, given that the Lane and Terry model is tested using the SEQ rather than a POMS derivative, hereafter we refer to dejection rather than depression because dejection is an item of the SEQ whereas depression is not.

A promising aspect of previous tests of Lane and Terry’s (2000) model has been the scale of affective and performance differences found between dejection groups. Extending this line of enquiry to include other salient variables may provide insight into the potential value of using dejection/no-dejection as a dichotomous variable in a broader range of investigations. A key feature of Lane and Terry’s model is the notion that dejection catalyses a negative self-schema that causes the recall of negative outcomes. The present study extended investigation to relevant aspects of personality such as trait emotion regulation, beliefs about emotion regulation, emotion regulation efficacy, and psychological skills usage across dejection and no-dejection groups. Given that emotions are transitory, identifying relationships with more stable personality constructs is appealing because they might predict how emotions fluctuate and can be regulated (LeBlanc et al., 2015). Trait emotion regulation is a particularly relevant personality trait (Gross and Thompson, 2007; Gross, 2015). The trait emotion regulation measure (Gross and John, 2003) has two factors: re-appraisal and suppression. Re-appraisal involves thinking differently about an emotion-eliciting situation and associates with positive behavior, health, and effective emotion regulation. Suppression involves reducing the response to an emotion-eliciting stimulus and typically associates with negative effects (Gross and Thompson, 2007; Gross, 2015). Re-appraisal is preferred to suppression because it facilitates early identification of situational factors that might trigger unpleasant or unhelpful emotions. Suppression tends to occur late in the emotion regulation process such as after a person has already become very angry instead of perhaps using re-appraisal to reframe the situational factors that caused the anger initially. In the context of Lane and Terry’s (2000) model, frequent use of suppression could be associated with dejection which, in turn, is associated with an intense unpleasant emotion profile. Use of re-appraisal may prevent unpleasant emotions occurring in the first place.

Perceived emotion regulation ability, emotion regulation efficacy (Kirk et al., 2008; Gross, 2015), and psychological skills usage (Thomas et al., 1999) are also relevant to emotion regulation. Individuals with successful emotion regulation experiences, who feel confident about regulating emotional states in the future, and who use psychological skills frequently, should be less likely to experience dejection. Further, frequent use of psychological skills (e.g., imagery, relaxation, self-talk) is associated with appraising anxiety as helpful for performance (Fletcher and Hanton, 2001), suggesting that those who actively engage in psychological skills training more effectively regulate emotional states.

Beliefs about the function and utility of emotions are central to the notion that emotions influence performance via learning rules (Baumeister et al., 2007; Hanin, 2010). Gross (2015) argued that learning rules help to explain contextual factors that influence emotion-performance relationships, such as confrontational tasks in which anger has been found to be facilitative of performance. Research has shown that individuals may learn to appraise unpleasant emotions as helpful and pleasant emotions as harmful, based on whether the emotions in question previously proved facilitative or debilitative of performance (Robazza and Bortoli, 2007; Jones et al., 2009; Hanin, 2010). Effort expenditure is also of interest in the present study. Increased effort is typically associated with better performance (Brinkmann and Gendolla, 2008; Lane et al., 2016). However, although effort might lead to better performance if attention remains focused on key performance cues, undue effort expended to regulate emotions might prove detrimental to performance (Muraven et al., 1998). Lane and Terry (2000) proposed that people use affective states as information about whether expending additional effort will result in goal attainment. In a dejected state, rather than initiating a search for solutions, accompanying emotions such as anger and anxiety tend to be directed toward negative self-thoughts, engendering a demotivating effect. In contrast, Lane and Terry argued that anxiety and anger can serve a functional role by signaling whether conditions warrant action and could provide a motivating effect if performance outcome is considered by the individual to be important enough to exert additional effort.

In the present study, we tested and extended Lane and Terry’s (2000) model using a very large sample of data collected as part of an online experiment (Lane et al., 2016). We tested seven hypotheses. The first hypothesis tested was that participants in the dejection group would report higher scores for unpleasant emotions and lower scores for pleasant emotions. Second, we hypothesized that interrelationships among emotions would be stronger in the dejection group than the no-dejection group. Third, we hypothesized that feeling energetic, excited, and happy would associate with good performance regardless of the presence or absence of dejection. Fourth, we hypothesized that anger and anxiety would show curvilinear relationships with performance in the no-dejection group but inverse linear relationships in the dejection group. Fifth, we hypothesized that dejection would be associated with inferior performance. Sixth, in an extension to Lane and Terry’s model, we hypothesized that dejection would be associated with lower scores for trait re-appraisal of emotion, emotional regulation ability, regulation efficacy, and lower usage of psychological skills, but higher scores for trait suppression of emotion. Seventh, we hypothesized that dejection would associate with a negative perception of performance wherein dejected participants would report lower scores for subjective performance and effort, and believe that their pre-competition emotional state was not helpful.

## Materials and Methods

### Participants

Participants were 73,588 volunteers recruited to the project via the British Broadcasting Corporation (BBC) Lab UK (Age: *M* = 34.5 year, *SD* = 14.0 year; Male = 46,839, Female = 26,698) with 51 participants not reporting gender. The website titled the project *Can You Compete Under Pressure?*^1^ which was presented by four-time Olympic champion Michael Johnson. An inclusion criterion was for participants to have indicated they were at least 18 years of age.

### Pre-competition Measures

#### Emotions

Emotions were assessed using the items, “Happy,” “Anxious,” “Dejected,” “Energetic,” “Angry,” and “Excited.” Five items were selected to reflect the same-named factors of the SEQ (Jones et al., 2005). One additional item, “Energetic,” was included to reflect arousal level. This addition was deemed important to better reflect the circumplex model of emotion that distinguishes between high and low-activation levels (Larsen and Diener, 1987). Each item was rated on a 7-point scale from 1 (“*not at all*”) to 7 (“*extremely*”) and participants responded to how they felt “right now.”

#### Emotion Regulation Strategies

Emotion regulation traits were measured using the Emotion Regulation Questionnaire (ERQ; Gross and John, 2003), a 10-item scale assessing habitual use of re-appraisal and suppression. Six items assessed re-appraisal usage [e.g., “When I want to feel more positive emotion (such as joy or amusement) I change what I’m thinking about”; “I control my emotions by changing the way I think about the situation I’m in”] and four items assessed suppression usage (e.g., “I keep my emotions to myself”; “When I am feeling negative emotions, I make sure not to express them”). Items were scored on a 7-point scale from 1 (*strongly disagree*), through 4 (*neutral*), to 7 (*strongly agree*). Alpha coefficients in the present study were acceptable for re-appraisal (α = 0.85) and suppression (α = 0.75).

#### Perceived Ability to Regulate Emotions

Perceived ability to regulate emotions was assessed using three items: “How successful are you at controlling your emotions?,” “How good are you at keeping your feelings under control?” (Niven et al., 2013) and “How confident are you in being able to change your emotions?” (Kirk et al., 2008). Items were scored on a 10-point scale from 1 (“*strongly disagree*”) to 10 (“*strongly agree*”). An acceptable alpha coefficient (α = 0.75) was found for the three items.

#### Regulation Efficacy

Regulation efficacy was assessed using the item “How confident are you in being able to get yourself mentally ready before performing?” which was developed for the purpose of the study from guidelines by Bandura (2006), who proposed that self-efficacy measures should assess key concepts directly, which in the present study was mental preparation to perform in a competitive task. The item was scored on a 10-point scale from 1 (“*not at all*”) to 10 (“*very confident*”).

#### Psychological Skills Usage

Psychological skill habits were assessed using eight competition-related items from the Test of Performance Strategies (TOPS; Thomas et al., 1999). Items were “During competition I don’t think about performing much; I just let it happen,” “During competition I play/perform instinctively with little conscious effort,” “I rehearse my performance in my mind at competitions,” “I imagine my competitive routine before I do it at a competition,” “I say things to myself to help my competitive performance,” “I manage my self-talk effectively during competition,” “I am able to relax if I get too nervous at competition,” and “When I need to, I can relax myself at competitions to get ready to perform” (α = 0.73). Items were scored on a 5-point scale from 1 (“*never*”) to 5 (“*always*”).

### Post-competition Measures

#### Mental Effort

The Rating Scale of Mental Effort (RSME; Zijlstra, 1993) is a single item scale that was used to assess mental effort. It assesses effort on a scale ranging from absolutely no effort (0) to complete effort (150). The RSME includes nine additional descriptive indicators (e.g., “some effort,” “extreme effort”) along the scale to assist participants to quantify their mental effort. The subjective nature of effort means that the relative reliability of the RSME is difficult to gauge as people invest different degrees of effort depending on task and personal requirements. The measure has been used previously in sport psychology research to assess effort in relation to emotions and performance in competition (Balmer et al., 2007).

#### Subjective Performance

The single item “How well did you perform?” was used to assess self-rated performance on a scale from 1 (“not at all well”) to 7 (“very well”).

#### Beliefs in the Influence of Emotions

The extent to which participants believed they had successfully managed their emotions during the game, was assessed via two items: “How successfully did you manage your emotions during the game?” and “Did your emotions help your performance?” both of which were rated from 1 (“not at all”) to 7 (“very much so”).

### Performance Task

The performance task was a competitive game that involved finding numbers in sequence from 1 to 36 as fast as possible from a 6 × 6 grid that was fully populated by the 36 numbers. Numbers were randomly assigned to the cells of the grid with no duplication. Participants competed against 1 of 12 different computer-simulated, ability-matched opponents generated from data collected in a pilot study (*n* = 300). Participants received a new random grid each time they completed the performance task. They could complete the grid using mouse or keyboard and were not informed that the opponent was ability-matched. The validity of the finish time was determined via examination of time stamps for each number identified. This allowed identification of lengthy delays between key strikes and therefore enabled identification of computer error or participants leaving the game. Internal consistency for the 36-items was α = 0.996, and α = 0.995 for the practice and competition rounds, respectively.

### Procedure

BBC Lab UK launched a publicity campaign to recruit participants via a promotional film and news features on prominent national television and radio programs. Data were collected online via the BBC Lab UK website^2^ over a 12-month period. The research was approved by the ethics committee of the School of Sport, Performing Arts and Leisure, at the University of Wolverhampton, UK. Participants provided written informed consent before proceeding. All participants registered with the BBC Lab UK prior to inclusion in the study.

First, participants reported basic demographic details and completed individual difference measures (emotion regulation strategies, perceived ability to regulate emotions, regulation efficacy, and psychological skills usage). Second, participants viewed a video in which Michael Johnson introduced the competition. Participants then reported pre-practice emotions before completing a practice round. Practice round scores were used to identify appropriate computer-generated opponents for the next attempt at the task. After completing the practice round, participants reported mental effort, subjective performance, and beliefs in the influence of emotions immediately post-task. Third, participants viewed a video in which Michael Johnson introduced the main performance task. Participants then reported pre-competition emotions before completing the main performance task, which involved direct competition against the computer-generated, ability-matched opponent. After completing the competitive task, participants reported post-task measures including performance satisfaction, beliefs about performance and effort exerted.

Data were cleaned before conducting the main data analysis following the guidelines of Tabachnick and Fidell (2013). Given the very large sample, a conservative approach to data cleansing was adopted. Errors in completion were minimal on a variable by variable basis but identifiable when multiple variables were examined. An example of a data entry error or score that was deemed implausible was someone responding with the most extreme score for each variable. Such multivariate outliers were identified using the Mahalanobis distances method and a total of 949 participants (∼1%) removed from the dataset.

Data were analyzed by first investigating the distribution of dejection scores. Lane and Terry (2000) found that approximately 50% of participants reported no symptoms of depressed mood. Hence, we first dichotomized participants into two dejection groups (dejection vs. no-dejection) prior to testing the Lane and Terry (2000) model. MANOVA was used to test hypothesis 1, that participants in the dejection group would report higher scores for unpleasant emotions and lower scores for pleasant emotions. Hypothesis 2, that interrelationships among emotions would be stronger in the dejection group than the no-dejection group, was tested by comparing internal consistency coefficients among discrete emotions in the two groups. Our use of items from the SEQ rather than the POMS to assess emotions precluded a direct test of hypothesis 3 as described in the Lane and Terry model. The SEQ is more focused on pleasant emotions compared to the focus on unpleasant emotions in the POMS and its derivatives. We used structural equation modeling to test a revised hypothesis 3 that feeling energetic, excited, and happy would associate with good performance regardless of the presence or absence of dejection. To test hypothesis 4, that anger and anxiety would show curvilinear relationships with performance in the no-dejection group but inverse linear relationships in the dejection group, two-factor ANOVAs (dejection × anger/anxiety) were used. Frequency analyses were used to group anger and anxiety scores into low, moderate, and high groups. We investigated differences in objective performance between the dejection and no-dejection group using an independent samples *t*-test. Hypotheses 5–7 were tested using MANOVA to compare dejection and no-dejection groups on objective performance, trait emotion regulation, emotion regulation ability, emotion regulation efficacy, psychological skills usage, effort exerted, subjective performance, and beliefs about the influence of pre-performance emotions.

## Results

Initial analysis indicated that 50,054 participants (69%) reported the lowest score (1 = not at all) on the dejection item. These participants made up the “no dejection” group. Conversely, 23,534 participants (31%) reported a score of 2 or higher on the scale and 14% of participants reported a score of 3 or higher on the 1–7 scale. Collectively, these participants were congregated into the “dejection” group.

In terms of performance time taken to complete the game, results indicated it was positively skewed with clustering for faster times and a long tail for slower times. This lack of normality was corrected using an inverse transformation (Box and Cox, 1964), which quantifies the rate at which participants completed the grid (i.e., 36 divided by completion time) whereby higher scores represent better performance.

### Hypothesis 1

In support of hypothesis 1, MANOVA indicated significant differences in emotional responses between the dejection and no dejection groups (Wilks lambda_5,72582_ = 0.72, *p* < 0.001, $\eta_{p}^{2}$ = 0.28). Dejection associated with lower scores for feeling energetic, excited, and happy, and higher scores for feeling anxious and angry (see **Table 1**).

**Table 1:** Comparison of emotions, perceived ability to regulate emotions, regulation efficacy, emotion regulation strategies, psychological skills usage, mental effort, subjective performance, and beliefs in the influence of emotions, between dejection and no-dejection groups.

	No dejection		Dejection		*F*^1,72586^	$\eta_{p}^{2}$
	(*n* = 50,054)		(*n* = 23,534)			
				
	*M*	*SD*	*M*	*SD*		
Happy	4.18	1.50	3.29	1.40	5637.71^∗^	0.072
Anxious	1.90	1.25	3.19	1.57	13901.43^∗^	0.161
Energetic	3.40	1.57	3.05	1.49	794.67^∗^	0.011
Angry	1.15	0.60	2.10	1.41	16132.13^∗^	0.182
Excited	3.24	1.69	2.93	1.58	532.89^∗^	0.007
Perceived ability to regulate emotions	6.53	1.62	6.11	1.65	1021.32^∗^	0.014
Emotion regulation self-efficacy	6.49	1.70	5.99	1.78	1333.69^∗^	0.018
Psychological skills usage	20.48	4.30	19.74	4.29	455.82^∗^	0.006
Re-appraisal	4.89	1.03	4.61	1.05	1069.03^∗^	0.015
Suppression	3.93	1.18	4.05	1.20	160.31^∗^	0.002
Mental effort	72.40	22.86	73.87	21.27	67.07^∗^	0.001
Subjective performance	3.91	1.68	3.58	1.65	595.30^∗^	0.008
Emotions managed successfully	4.49	1.68	4.04	1.60	1195.86^∗^	0.016
Emotions helped performance	3.26	1.79	3.18	1.67	25.56^∗^	0.000


*^∗^*p* < 0.0001.*

### Hypothesis 2

Contrary to hypothesis 2, the average inter-item correlation for the two dejection groups did not differ significantly (no-dejection group: α = 0.61; dejection group: α = 0.51). As shown in **Table 2**, intercorrelations among emotions were in the same direction and of similar magnitude in both groups.

**Table 2:** Intercorrelations among emotions in the no-dejection and dejection groups.

	No-dejection				Dejection			
		
	Anxious	Energetic	Angry	Excited	Anxious	Energetic	Angry	Excited
Happy	-0.06	0.52^∗^	-0.13^∗^	0.49^∗^	-0.09	0.51^∗^	-0.21^∗^	0.50^∗^
Anxious	1.00	0.07	0.11^∗^	0.16^∗^	1.00	0.03	0.25^∗^	0.08
Energetic		1.00	-0.02	0.67^∗^		1.00	-0.05	0.66^∗^
Angry			1.00	-0.02			1.00	-0.04


*^∗^*p* < 0.01.*

### Hypothesis 3

Multi-group structural equation modeling to test hypothesized relationships between emotional responses and performance in the two dejection groups indicated a good fitting model (*x*^2^ = 28.702, *df* = 5, *p* < 0.001, CFI = 1.000, RMSEA = 0.011). Emotions predicted 2% of performance variance in the no-dejection group and 3% of performance variance in the dejection group. In partial support of hypothesis 3, feeling excited and happy significantly facilitated performance in both groups whereas feeling energetic was unrelated to performance, regardless of the presence or absence of dejection. Lagrange Multiplier Test scores confirmed that the relationship between anxiety and performance differed among dejection groups (*x*^2^ = 13.053, *p* < 0.001), with anxiety showing a marginally stronger positive relationship with performance in the dejection group (see **Table 3**).

**Table 3:** Multi-Group Structural Equation Modeling of Emotion-Performance Relationships in the no-dejection and dejection groups.

	No-dejection Standardized *r*	Dejection Standardized *r*
Happy	0.012^∗^	0.011^∗^
Anxious	0.010^∗^	0.013^∗^
Energetic	-0.006	-0.005
Angry	-0.016^∗^	-0.037^∗^
Excited	0.035^∗^	0.033^∗^


*^∗^*p* < 0.001.*

### Hypothesis 4

Hypothesis 4 was partially supported, with dejection showing a significant moderating effect for anxiety scores but not for anger scores. A two-factor (dejection × anger) ANOVA showed significant main effects for dejection (*F*_1,72582_ = 10.334, *p* < 0.001, $\eta_{p}^{2}$ = 0.0004) and anger (*F*_1,72582_ = 14.33, *p* < 0.001, $\eta_{p}^{2}$ = 0.0001) but did not identify the hypothesized significant interaction effect (*F*_1,72582_ = 1.70, *p* > 0.01). High anger scores associated with worse performance in both dejection groups (*p* < 0.001).

**FIGURE 2:**
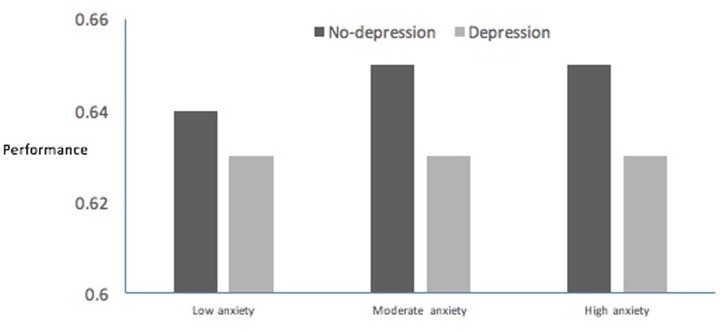
**Anxiety and performance relationships for dejection and no-dejection groups**.

For anxiety, a two-factor (dejection × anxiety) ANOVA showed significant main effects for dejection (*F*_1,72582_ = 76.05, *p* < 0.001, $\eta_{p}^{2}$ = 0.001) and anxiety (*F*_1,72582_ = 9.69, *p* < 0.001, $\eta_{p}^{2}$ = 0.0003), and confirmed the hypothesized significant interaction effect (*F*_1,72582_ = 6.30, *p* = 0.002, $\eta_{p}^{2}$ = 0.0002). The interaction effect indicated that high and moderate anxiety associated with better performance than low anxiety in the no-dejection group but not in the dejection group (**Figure 2**).

### Hypothesis 5

Hypothesis 5 was supported, with results confirming that the no-dejection group (*M* = 0.65, *SD* = 0.18) significantly outperformed the dejection group (*M* = 0.63, *SD* = 0.18) by 3.2% (*t*_72586_ = 8.42, *p* < 0.0001, *d* = 0.11).

### Hypothesis 6

Hypothesis 6 was supported. MANOVA to compare emotion regulation traits, emotion regulation beliefs, regulation efficacy, use of psychological skills, intensity of effort exerted, beliefs about the quality of mental preparation, and satisfaction with performance on the concentration task showed a significant effect of dejection (Wilks lambda_9,72578_ = 0.95, *p* < 0.001, $\eta_{p}^{2}$ = 0.048). The dejection group reported significantly lower scores for re-appraisal and higher scores for suppression. Dejection also associated with lower scores for perceived ability to regulate emotions, regulation self-efficacy, and lower usage of psychological skills (see **Table 1**).

### Hypothesis 7

Hypothesis 7 was partially supported, with the dejection group reporting significantly lower scores for subjective performance, lower perceptions that emotions helped them perform better, and lower scores for the belief that emotions were managed successfully (**Table 1**). However, counter to the hypothesis, dejected participants reported higher scores for exerting mental effort.

## Discussion

The present study evaluated Lane and Terry’s (2000) conceptual model using a large sample in the context of an online competitive task. We tested the model using a valence-balanced measure of emotion (Jones et al., 2005) rather than the more negatively valenced measure used in previous tests of the model. We extended the model by examining relationships between dejection and perceived ability to regulate emotions, regulation efficacy, emotion regulation strategies, psychological skills usage, mental effort, subjective performance, and beliefs in the management and influence of emotions. Given the very large number of participants involved, the present study represents a comprehensive evaluation and significant extension of Lane and Terry’s model, which has previously been shown to have practical and theoretical value (Lane and Terry, 2016).

Consistent with previous tests of the model (Lane and Terry, 2005) results showed that dejection associated with a generally unpleasant psychological state, supporting the first hypothesis (**Table 1**). This finding suggests that dejection is a viable alternative item to depression in investigations of emotional responses. Results did not support the second hypothesis, that discrete emotions would show stronger intercorrelations among participants reporting some level of dejection (**Table 2**). Lane and Terry (2000) argued that the negative schema associated with dejection acted as a catalyst for other negatively valenced emotions and so intercorrelations among, for example, confusion, fatigue, anger, and tension would tend to be high. In previous tests of the model, emotion has been assessed using the BRUMS (Terry et al., 1999, 2003), which has five unpleasant states with vigor as the only pleasant emotion. Vigor has tended to show weak inverse relationships with other emotions regardless of the presence or absence of depression (Lane and Terry, 2016). In the present study, use of the SEQ meant that subscales were more balanced in valence terms, which may explain why intercorrelations among emotions were weaker. The second hypothesis of the Lane and Terry model might be better rephrased to refer to unpleasant emotions only.

In terms of the third and fourth hypotheses, results showed that emotion-performance relationships were statistically significant but explained only 2–3% of performance variance and that dejection moderated relationships with performance for anxiety but not for anger (**Table 3** and **Figure 2**). The limited performance variance explained by emotions was lower than anticipated. Hanin (2010) has stressed the highly individualized nature of emotion-performance relationships, and has shown strong relationships using ideographic designs, especially in competition environments where participants had considerable prior experience. Given that a novel task was used in the present study, the emotional profile associated with good performance was not well established *a priori* for any individual participant, which may have served to reduce the impact of emotions on performance.

Methodological factors could also help to explain weak emotion-performance relationships. Terry (1995) identified conditions likely to strengthen the relationship between emotions and performance, including short duration events, a self-referenced performance criterion, and relatively homogeneous levels of ability. Although the first two conditions were met, significant heterogeneity in ability levels was evident, which may have served to confound relationships between emotions and performance. Also, the online environment in which data were collected needs to be considered. Online data collection affords the capture of large datasets but inevitably the conditions in which testing occurs will vary, potentially introducing additional confounds that may have weakened emotion-performance relationships. It should be noted, however, that dejection was shown to have a significant deleterious effect on performance (hypothesis 5).

Concerning the direction of specific emotion-performance relationships, Lane and Terry (2000) proposed that vigor would associate with better performance, although present results showed that feeling energetic (part of the vigor construct) was not significantly associated with better performance whereas other pleasant emotions (i.e., excitement and to a lesser extent happiness) significantly associated with better performance albeit weakly (**Table 3**). The lack of a significant relationship between feeling energetic and subsequent performance is likely explained by the nature of the performance task, which involved little physical exertion. That is, we would not expect the task to elicit an emotional response that would prompt individuals to feel energetic if the task is primarily cognitive with minimal fine motor movements. In other words, there was nothing inherent in the competition situation to trigger an appraisal that increased physical exertion would enhance performance.

For anger and anxiety, results offer partial support for Lane and Terry’s (2000) fourth hypothesis. As **Figure 2** indicates, high and moderate anxiety associated with better performance than low anxiety in the no-dejection group, although the effect size was small. In the dejection group, anxiety showed no relationship with performance. No support was found for differences in the strength and direction of anger-performance relationships between dejection groups.

Overall, results showed strong support for hypothesis 1, and partial support for hypotheses 3 and 4 of the Lane and Terry (2000) model. Using dejection as a dichotomous variable and evaluating its influence on other emotions appears to be worthwhile when investigating emotion-performance relationships. Participants who report dejection tend to report a generally negative emotional profile and among those reporting no dejection, anxiety could be functional for activities such as the short duration concentration task used in the present study.

Perhaps the most important finding from the present study is that dejection relates to a constellation of relevant constructs in a predictable way. Consistent with hypothesis 6, dejection was associated with greater use of suppression and reduced use of re-appraisal (Gross and Thompson, 2007), with lower scores for emotion regulation ability (Niven et al., 2013), lower emotional self-efficacy (Kirk et al., 2008), and less frequent use of psychological skills. Dejection was also associated with exerting greater effort (Zijlstra, 1993), low satisfaction with performance, and negative beliefs that emotions helped performance (Baumeister et al., 2007; Hanin, 2010, see **Table 1**). Thus, individuals who tend to use maladaptive strategies were more likely to report feeling dejected. Re-appraisal is proposed to be superior to suppression as a coping strategy as it anticipates potentially unwanted emotions (Webb et al., 2012).

Positive beliefs in being able to regulate emotions are proposed to be important in the process of effective emotion regulation (Lane et al., 2012). Using psychological skills to, for example, visualize successful performance can be considered to be a form of re-appraisal (Lane et al., 2012) and such usage was higher in the no-dejection group. Previous research has found that frequent use of psychological skills is associated with appraising anxiety as helpful for performance (Fletcher and Hanton, 2001). Psychological skills such as imagery, self-talk, and goal-setting can all be used to re-appraise a situation, thereby increasing self-belief and creating a more positive emotional profile (Hanin, 2010). Overall, the no-dejection group reported greater use of re-appraisal, confidence to regulate emotions, and greater use of psychological skills. In contrast, use of suppression to regulate dejection and other negative emotional states requires effort, which may direct attention away from performance thereby producing a detrimental effect (Muraven et al., 1998).

When participants report feeling dejected, the associated emotional state tends to be unpleasant and intense, effort invested tends to be high, and satisfaction with performance tends to be low. If emotions contribute to learning rules that guide behavior (Baumeister et al., 2007; Hanin, 2010), then feeling dejected will signal the need to regulate emotions. Therefore, when individuals experience such emotions in future, attempts at regulation will be initiated to reduce the gap between how they are feeling and how they wish to feel.

The finding that the dejection group exerted greater mental effort warrants attention. We hypothesized that dejection would associate with poor performance underpinned by low effort scores but results did not support this notion. Lane and Terry (2000) proposed that dejection influenced performance by serving an informational role that subsequently triggered action. Given the evidence that mild depressive states promote increased effort in competitive tasks (Brinkmann and Gendolla, 2008), dejected participants may have attempted to perform well by recognizing undesirable emotions and regulating them via increased mental effort (Gross and Thompson, 2007; Gross, 2015). An alternative explanation is that dejected participants perceived the same level of performance as more effortful than participants who are not dejected. The superior objective performance of the no-dejection group is consistent with this latter interpretation. Previous research has found that when participants report intense unpleasant emotions the same level of objective performance is achieved, but participants perceive performance to feel harder (Beedie et al., 2012). Importantly, Beedie et al. (2012) found dejection associated with increased lactic acid and higher oxygen uptake, suggesting that intense unpleasant emotions and increased effort might be underpinned by biological differences. Future research should consider assessing physiological markers of activity alongside perceptions of effort to gain a fuller assessment and facilitate examination of the potential influence of dejection.

Although the present study demonstrated the negative influence of dejection on psychological states and performance, some limitations should be acknowledged. First, although online research enables investigators to reach large audiences, the lack of control inherent in online data collection increases the potential for statistical noise. Replication of the same experiment under controlled conditions would illuminate the generalizability of the present findings. Second, with such a large sample, overpowered analyses and small observed effects, the probability of Type II errors is increased. Further data mining in the form of re-analysis of one or more data subsets chosen randomly from the overall dataset might be advantageous. Third, effect sizes for emotion-performance relationships were small, suggesting that some moderators may not have been adequately controlled. Illuminating the overview picture by interrogating a very large intact dataset was an important and necessary initial step, but future research might explore the dataset further to identify the strength of moderators influencing emotion-performance relationships. However, as previous research has identified, emotion-performance relationships tend to be individualized (Terry, 1995; Hanin, 2010; Lane and Terry, 2016) and cross-sectional studies will always be inherently limited when it comes to explaining a large proportion of performance variance from measures of emotion.

In terms of theoretical developments, our findings suggest that the Lane and Terry (2000) model is in need of revision. Lane and Terry based their conceptual model on the measurement model of the POMS, primarily because it was the measure of choice for researchers rather than for compelling theoretical reasons. In the present study we assessed a larger number of pleasant emotional states, showing that dejection associated with lower self-reports of energy, excitement and happiness, which is consistent with the linear effects of depression on unpleasant emotions reported in previous tests of the model (Lane and Terry, 2005). The most consistent finding from tests of Lane and Terry’s model is that dichotomising dejection into two groups provides an insightful method of analyzing and interpreting self-report data. Present findings add support to the notion that dejection (or depression) associates with a negative profile on all self-report measures used, including other emotions, psychological skills, trait measures and satisfaction with performance.

We recommend that Lane and Terry’s model be extended by the addition of other theory-led variables. A benefit of theory-led research over exploratory investigations is to help delimit studies and thereby reduce the likelihood of the researcher being overwhelmed by the complexity of the subject matter. Future tests of a revised model should establish specific and testable hypotheses and, importantly, propose the mechanism(s) by which dejection is influential. This work is challenging and was not feasible within the scope of the present study. We encourage researchers to look beyond self-report for the assessment of the antecedents, correlates, and effects of dejection. Physiological variables associated with dejection and no-dejection groups and how these relate to the effort exerted and subsequent performance should be considered (see Beedie et al., 2012). Identifying relationships with physiological variables would offer insight into how emotions might influence performance. In the present study, we cannot be sure if the higher effort scores among the dejection group reflect objectively greater effort or simply reflect perceptions that effort was greater.

## Conclusion

Although relatively few people experienced substantial dejection in the competitive setting under investigation, where people reported even minor levels of dejection it had a negative impact on their overall emotional profile and other psychological states. In such instances, it appears that people reported the belief that their psychological state was not helpful. Research that examines coping with feeling dejected is scant, but is of intuitive value to applied theorists and practitioners. The present study indicates that feeling dejected was associated with using maladaptive emotion regulation strategies such as suppression rather than adaptive strategies such as re-appraisal. It also appears that participants sought to regulate dejection by increasing effort. Given evidence suggesting detrimental effects of dejection, we recommend that future research continues to investigate the role of unwanted emotions in emotion regulation and performance, and identifies effective ways of coping with dejection.

## Ethics Statement

The project was approved by the ethics committee of the University of Wolverhampton and later endorsed by the University of Sheffield. All participants gave informed consent in accordance with the Declaration of Helsinki. The study was carried out in accordance with the recommendations of the British Psychological Society and British Association of Sport and Exercise Sciences.

## Author Contributions

The online project from which the main data set was collected was developed principally by AL and PaT. The paper develops the Lane and Terry (2000) conceptual model and Lane played a central role in writing, analyzing, and interpreting the data. PT insight and knowledge helped shape the paper and give clarity to the ideas presented. During the development of the project and the writing of this paper TD and AF made meaningful contribution to the development and revision of ideas. PcT made a substantial contribution to the development of the idea to test Lane and Terry’s model, writing the paper, interpreting the results and discussing the findings.

## Conflict of Interest Statement

The authors declare that the research was conducted in the absence of any commercial or financial relationships that could be construed as a potential conflict of interest.

## Footnotes

1 http://www.bbc.co.uk/labuk/experiments/can-you-compete-under-pressure

2 http://www.bbc.co.uk/guides/zwr2mnb
